# Self-harm, early emotional neglect and alexithymia in young Italian help seekers

**DOI:** 10.1192/j.eurpsy.2025.655

**Published:** 2025-08-26

**Authors:** S. Mammarella, L. Giusti, S. Del Vecchio, A. Salza, M. Casacchia, R. Roncone

**Affiliations:** 1 Department of Life, Health and Environmental Sciences, University of L’Aquila, L’Aquila, Italy

## Abstract

**Introduction:**

Recent studies showed that non-suicidal Self-Injury (NSSI) is a transdiagnostic behavior with multiple functions and motivational factors. More specifically, two key functions have been identified: the first is an interpersonal function (hurting/punishing others, influencing others’ behavior); the second involves personal goals (emotions regulation, dissociation, self-punishment). Some distal factors may contribute to the onset of this behavior (e.g., childhood abuse, emotional neglect), resulting in emotional dysregulation and alexithymia. Alexithymia, also called emotional blindness, a coping strategy to reduce emotional pain following trauma traumatic life events, emerged as a predictor of NSSI.

**Objectives:**

The study aimed to investigate in a sample of young Italian help seekers referred to an early intervention service and a university counseling and consultation service (1) the prevalence of NSSI behaviors; (2) their correlation to early traumatic life experiences and alexithymia symptoms.

**Methods:**

From March 2023, the young adults participating in this study filled a standardized battery (through a QR code). In this preliminary analysis, only data concerning the Deliberate Self-Harm Inventory (DSHI), Childhood Trauma Questionnaire – Short Form (CTQ-SF), and the Toronto Alexithymia Scale (TAS – 20) were considered.

**Results:**

Nowadays, 73 young adults participated in the study (60% women; mean age 26 years, SD 4.2). A third of the sample reported NSSI behavior lifetime, with a higher distribution among women (χ2= 9.425 p=0.002). By comparing the NSSI and No-NSSI groups, only the emotional neglect dimension exceed the clinical cut-off even if statistically significant differences emerged in all the early traumatic dimensions, with higher scores in the NSSI group as regard the emotional abuse (F= 18.321; p=0.000), physical abuse (F= 17.556; p=0.000), sexual abuse (F= 5.200; p=0.026), emotional neglect (F= 20.053; p=0.000), physical neglect (F= 12.134; p=0.001), minimization/denial (F= 13.384; p=0.000). The alexithymia symptoms, assessed with the TAS-20 scale, the NSSI group showed higher scores with a statistically significant difference compared to the No-NSSI group (F= 15.842; p=0.000) and, specifically, in the “*difficulty describing feelings*” (F= 10.351; p=0.002) and “*difficulty in identifying emotions*” (F= 13.543; p=0.000).

**Conclusions:**

According to the social neuroscience model, these preliminary results suggested that young adults that practice self-harm behaviors may have experienced early emotional neglect, determining a vulnerability to recognize and regulate emotions in response to stressful events. It is therefore crucial to assess and monitor this behavior in all young people help seekers from mental health services, concerning the history of traumatic events and emotional difficulties, to plan personalized evidence-based interventions.

**Disclosure of Interest:**

None Declared

